# Herd effect from influenza vaccination in non-healthcare settings: a systematic review of randomised controlled trials and observational studies

**DOI:** 10.2807/1560-7917.ES.2016.21.42.30378

**Published:** 2016-10-20

**Authors:** Dominik Mertz, Shaza A. Fadel, Po-Po Lam, Dat Tran, Jocelyn A Srigley, Sandra A Asner, Michelle Science, Stefan P Kuster, Johannes Nemeth, Jennie Johnstone, Justin R Ortiz, Mark Loeb

**Affiliations:** 1 Department of Medicine, McMaster University, Hamilton, Canada; 2 Department of Clinical Epidemiology and Biostatistics, McMaster University, Hamilton, Canada; 3 Department of Pathology and Molecular Medicine, McMaster University, Hamilton, Canada; 4 Michael G. DeGroote Institute for Infectious Diseases Research, McMaster University, Hamilton, Canada; 5 Centre for Global Health Research, Li Ka Shing Knowledge Institute, St. Michael’s Hospital, Toronto, Canada; 6 Dalla Lana School of Public Health, University of Toronto, Toronto, Canada; 7 Division of Infectious Diseases, Department of Paediatrics, The Hospital for Sick Children, University of Toronto, Toronto, Canada; 8 Department of Laboratory Medicine, BC Children’s & Women’s Hospital, Vancouver, Canada; 9 Pediatric Infectious Diseases Unit, Department of Pediatrics, University Hospital Lausanne, Lausanne, Switzerland; 10 Infectious Diseases Service, Department of Medicine, University Hospital Lausanne, Lausanne, Switzerland; 11 Division of Infectious Diseases and Hospital Epidemiology, University Hospital and University of Zurich, Zurich, Switzerland; 13 Public Health Ontario, Infection Prevention and Control, Toronto, Canada; 14 St. Joseph’s Health Centre, Toronto, Canada; 15 Department of Medicine, University of Toronto, Toronto, Canada; 16 Initiative for Vaccine Research, World Health Organization, Geneva, Switzerland

**Keywords:** influenza, vaccines and immunisation, vaccine-preventable diseases, herd effect, indirect effect, systematic review

## Abstract

Influenza vaccination programmes are assumed to have a herd effect and protect contacts of vaccinated persons from influenza virus infection. We searched MEDLINE, EMBASE, the Cumulative Index to Nursing and Allied Health Literature (CINAHL), Global Health and the Cochrane Central Register of Controlled Trials (CENTRAL) from inception to March 2014 for studies assessing the protective effect of influenza vaccination vs no vaccination on influenza virus infections in contacts. We calculated odds ratios (ORs) and 95% confidence intervals (CIs) using a random-effects model. Of 43,082 screened articles, nine randomised controlled trials (RCTs) and four observational studies were eligible. Among the RCTs, no statistically significant herd effect on the occurrence of influenza in contacts could be found (OR: 0.62; 95% CI: 0.34–1.12). The one RCT conducted in a community setting, however, showed a significant effect (OR: 0.39; 95% CI: 0.26–0.57), as did the observational studies (OR: 0.57; 95% CI: 0.43–0.77). We found only a few studies that quantified the herd effect of vaccination, all studies except one were conducted in children, and the overall evidence was graded as low. The evidence is too limited to conclude in what setting(s) a herd effect may or may not be achieved.

## Introduction

Influenza is a major cause of morbidity and mortality worldwide [1-3]. Many countries recommend vaccination against influenza to prevent influenza infections, in particular for groups at high risk for complications [4-7]. Some high risk groups, such as young children and elderly persons (commonly defined as those above 65 years of age), experience decreased influenza vaccine effectiveness compared with healthy adults [8,9], complicating influenza prevention strategies. Moreover, because such groups represent a minority of the population at large, the population-wide impact of vaccination of risk groups may be limited [7,10].

Influenza vaccine modelling and ecological studies identifying benefits of herd effect have informed seasonal and pandemic influenza vaccine policies [10,11], herd effect being usually defined as the indirect protection of individuals susceptible to infection when a sufficient proportion of the population is immune to the pathogen. Vaccinating persons most likely to respond to the influenza vaccine and relying on herd effect to reduce the chance of exposure to influenza may protect unvaccinated or high-risk individuals. Herd effect may therefore mitigate the consequences of impaired vaccine response in some high-risk groups [12-14].

The purpose of this systematic review was to summarise the evidence on herd effect from influenza vaccination outside healthcare settings. These data may help to inform public health on influenza vaccine research and policy development.

## Methods

All decisions regarding eligibility criteria, search strategy, study selection, assessment of risk for bias, explanation for heterogeneity, data collection and analysis were established before data collection. The protocol was registered with the international prospective register of systematic reviews (PROSPERO) [15] (CRD42014009401) and was reported in accordance with the PRISMA statement [16].

### Eligibility criteria and outcomes assessed

Studies assessing the protective effect of influenza vaccination vs no influenza vaccination (either no vaccination, placebo or alternative vaccine) on contacts of any age group in a non-healthcare setting were eligible. The definition of contacts was broad and included anyone in the same community, school or household. Study designs included randomised controlled trials (RCTs) and observational studies with a non-influenza vaccine comparator group. For the latter study type, quasi-experimental (before–after) studies, cohort studies, case–control studies and cross-sectional studies were eligible. Ecological studies and modelling studies were excluded. We also excluded studies conducted within healthcare institutions, such as nursing homes and hospitals, and studies in languages other than English.

The primary outcome was influenza in non-vaccinated contacts exposed to persons vaccinated against influenza vs those not vaccinated. Influenza included both laboratory-confirmed influenza (defined by one or more of the following: nucleic acid amplification testing, viral culture, antigen detection, pre-/post-season or acute/convalescent serology) or non-laboratory-defined evidence. Non-laboratory-defined evidence required the presence of influenza-like illness (ILI, as per the study definition) within a period of time when laboratory-confirmed influenza was circulating in the study area. Secondary outcomes included hospitalisation, pneumonia and death.

### Search strategy, study selection and data extraction

We searched MEDLINE (since 1950), EMBASE (since 1980), the Cumulative Index to Nursing and Allied Health Literature (CINAHL) (since 1982), Global Health (since 1973) and the Cochrane Central Register of Controlled Trials (CENTRAL) up to 7 March 2014. We also searched reference lists of identified articles and those of review articles for eligible studies.

Multiple teams of two reviewers independently screened titles and abstracts and, for studies identified by at least one reviewer to be of potential interest, full-text articles were screened. Data from eligible studies were extracted independently by two reviewers using a database. Any disagreement between the reviewers was resolved by consensus or arbitration by a third reviewer. We attempted to contact the first and corresponding author of the original article whenever potentially important information was missing.

Assessment of the risk of bias and of the overall quality of evidence was also conducted by two reviewers independently. We used the Cochrane Review Collaboration’s tool [17] to assess the risk of bias for RCTs, and the Newcastle-Ottawa scale (NOS) [18] to assess the quality of observational studies. The overall quality of evidence was assessed using the grading of recommendations assessment, development and evaluation (GRADE) criteria [19]. Given the small number of studies, no formal assessment of the risk of publication bias could be conducted [20].

### Data analysis

We performed meta-analyses of RCTs and observational studies separately. We calculated odds ratios (ORs) and corresponding 95% confidence intervals (CIs) as summary estimates using random-effects modelling (using RevMan 5.3 [21]).

We planned a priori to conduct two subgroup analyses. First, we examined herd effect by study setting, comparing the effect in household studies, school-based studies (where the impact on non-vaccinated schoolchildren was measured) and community studies. For community studies, those comparing geographically defined areas with different vaccination strategies were considered. We hypothesised that the closer the contact was to vaccinated persons, the stronger the effect would be. Second, we assessed whether the herd effect of the vaccination in young children (up to 5 years of age) was different from that in older children and teenagers (5–18 years), and in adults.

Heterogeneity was evaluated using χ^2^ and I^2^ statistics [22]. We considered a χ^2^ of < 0.10 or an I^2^ statistic of > 50% to reflect significant heterogeneity. If significant heterogeneity was found, we planned to perform additional subgroup analyses. Our a priori hypotheses to explain heterogeneity beyond the planned subgroup analyses were: laboratory-confirmed vs non-laboratory-confirmed influenza cases, and cases confirmed by nucleic acid amplification testing and viral culture vs cases confirmed by other laboratory methods. We also analysed the predominant circulating type/subtype (influenza A(H3N2) orA(H1N1), and influenza B).

## Results

**Figure 1:**
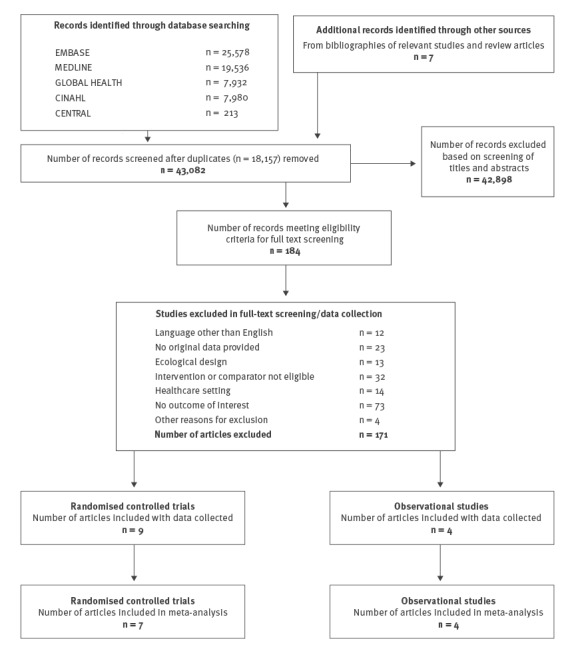
Flowchart of included and excluded randomised control trials and observational studies identified in a systematic review of herd effect from influenza vaccination in non-healthcare settings

^a^ Two randomised control trials did not report all numerator and denominator data and therefore could not be included in the meta-analysis.

After removing 18,157 duplicates, we screened a total of 43,082 titles and abstracts, reviewed 184 full-text articles and included nine RCTs and four observational studies in our systematic review (Figure 1). Of the 13 RCTs and observational studies, seven were conducted in North America, and two each in Italy and Russia, and one in Malaysia and Hong Kong Special Administrative Region, respectively (Table 1).

**Table 1:** Study characteristics of studies included in a systematic review of herd effect arising from influenza vaccination in non-healthcare settings

First author [source]	Study location	Study period	Predominant influenza virus type or subtype	Intervention group	Setting	Number of vaccinees	Number of contacts^a^	Laboratory confirmation of influenza
Randomised control trials								
Gruber [29]	United States	1985/86	B	Children aged 3–18 years	Household	133	123	Yes
Clover [33]	United States	1986/87	A(H1N1)	Children aged 3–19 years	Household	194	177	Yes
Rudenko^b^ [23]	Russia	1989–91	A(H3N2)	Children aged 7–14 years	School	11,071	Not available	No
Hurwitz [13]	United States	1996/97	Influenza B	Children aged 2–5 years	Household	127	228	No
Esposito [34]	Italy	2000/01	H1N1	Children aged 0.5–9 years	Household	127	349	No
Principi^b^ [24]	Italy	2001/02	Influenza B	Children aged 0.5–5 years	Household	303	1,098	No
Hui [31]	Malaysia	2005	Not reported	Adults aged 18–64 years	Household	346	362	No
Cowling [30]	Hong Kong SAR	2008/09	A(H3N2)	Children aged 6–15 years	Household	119	312	Yes
Loeb [12]	Canada	2009	A(H3N2)	Children aged 1.5–15 years	Community	947	2,326	Yes
Observational studies (all cohort studies)								
Piedra [26]	United States	1998–2001	A(H3N2)	Children aged 1.5–18 years	Community	ca 40,000	350,296	No
Ghendon [25]	Russia	2001–03	A(H3N2)	Children aged 3–17 years	Community	87,221	158,451	No
King [14]	United States	2004/05	A(H3N2)	Children aged 5–14 years	Household	2,717	3,022^c^	No
Kjos [27]	United States	2010/11	A(H3N2)	Children, age unavailable	Elementary school (5–10 year-olds)	1,012	937	No

SAR: Special Administrative Region.

^a^ The definition of contacts was broad and included anyone in the same community, school or household.

^b^ The randomised control trial did not report all numerator and denominator data and therefore could not be included in the meta-analysis.

^c^ In this study, the number of contacts was not reported. The number shown is the number of households (3,022) included in the analysis in intervention schools; there were 5,488 households in control schools).

### Findings from randomised controlled trials

Of the nine RCTs included, seven were conducted in a household setting, one in a school and one in a community setting (Table 1). The intervention group consisted of children in all but one study. The total sample size of contacts was 4,975, with one study –the largest– not reporting the total number of contacts [23].

**Figure 2:**
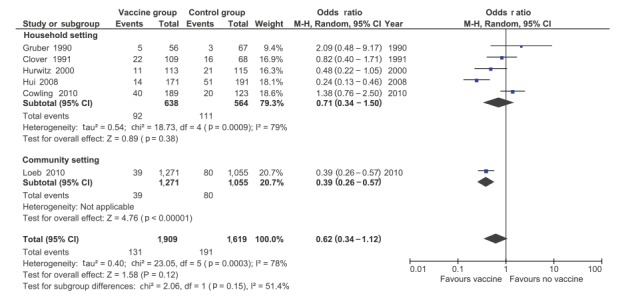
Meta-analysis of seven included randomised controlled trials reporting on influenza infections in contacts of influenza vaccinated vs unvaccinated individuals in non-healthcare settings

CI: confidence interval; df: degrees of freedom; M-H: Mantel-Haenszel.

A total of six RCTs provided data for the primary analysis comparing influenza-like illness in contacts of vaccinated vs unvaccinated persons (Figure 2). Overall, no statistically significant herd effect was found (OR: 0.62; 95% CI: 0.34–1.12), with significant statistical heterogeneity (I^2^ = 78%). Only one study, by Loeb et al., assessed contacts for influenza virus infection at community level: vaccination of children reduced the influenza infection rate for the community (OR: 0.39; 95% CI: 0.26–0.57) [12]. In contrast, there was no statistically significant effect in the subgroup of RCTs assessing household contacts (OR: 0.71; 95% CI: 0.34–1.50). No other differences between subgroups were found (p = 0.15 for subgroup differences). There was an 86% reduction in the odds of 5–17 year-old contacts of vaccinated individuals becoming infected as compared with contacts of unvaccinated individuals (OR: 0.14; 95% CI: 0.03–0.70), while no statistically significant differences were found when contacts were less than five years-old or adults. This difference across age groups was not statistically significant (p = 0.26).

Given the significant amount of statistical heterogeneity in the primary analyses, we conducted additional subgroup analyses. Subgrouping by whether or not influenza was laboratory confirmed did not significantly reduce statistical heterogeneity (p for subgroup differences was 0.06; I^2^ = 70·8%), with a significant effect on influenza infections in contacts in RCTs with no laboratory confirmation (OR: 0.33; 95% CI: 0.17–0.64; I^2^ = 43%; n = 2) and no effect in RCTs using laboratory confirmation (OR: 0.87; 95% CI: 0.40–1.89; I^2^ = 81%; n = 4). Subgrouping by type of laboratory confirmation or by influenza virus type/subtype could not further explain the statistical heterogeneity.

Two RCTs provided data on hospitalisation of contacts, with no statistically significant difference seen (OR 0.83; 95% CI: 0.17–4.1). Only the RCT by Loeb et al. [12] reported on mortality and pneumonia in contacts, with no effect of the vaccine on either of these outcomes in community contacts. Because of the limited number of studies reporting these outcomes, no subgroup analyses could be performed.

Two other RCTs demonstrated a herd effect of influenza vaccination, but the data provided in the publications did not report the numerators and denominators needed for our meta-analysis, and we were unable to obtain further data or information from the authors. Principi et al. concluded that influenza vaccination significantly reduced the direct and indirect influenza-related costs in healthy children and their unvaccinated family members [24]. Rudenko et al. found that the use of a live attenuated influenza vaccine was associated with a lower rate of influenza-like illness in school staff and non-vaccinated children when comparing schools that had vs schools that did not have an institutional influenza vaccination programme [23].

### Findings from observational studies

A total of four observational studies were identified (Table 1). The intervention groups consisted of children in all the studies. Two studies were conducted in a community setting, and one each in the household and school setting. The total sample size of contacts was more than 500,000. The level of analysis was the household, and not the individual person, in one of the studies [14].

**Figure 3:**
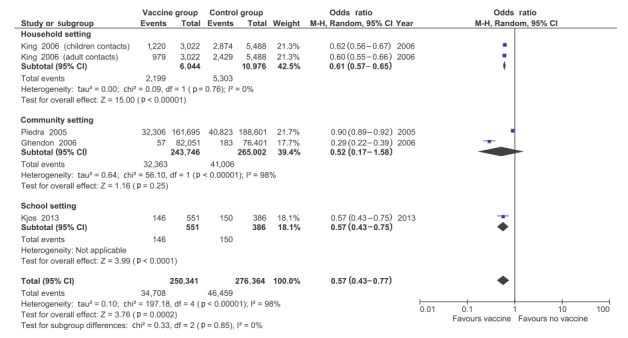
Meta-analysis of four included observational studies reporting on influenza infections in contacts of influenza vaccinated vs unvaccinated patients in non-healthcare settings

CI: confidence interval; df: degrees of freedom; M-H: Mantel-Haenszel.

Meta-analysis showed a significant reduction of influenza illness in contacts of vaccinated patients (OR 0.57; 95% CI: 0.43–0.77) (Figure 3). Heterogeneity was very high (I^2^ = 98%); however, the direction of the effect was identical in all studies, only the amount of the effect size varied across studies. No age-specific data were available. When comparing the three study settings, no significant subgroup effect was found (p = 0.85 for subgroup differences). Given that all studies were lacking laboratory confirmation, and all were conducted during influenza A(H3N2)-predominant influenza seasons, no further subgroup analyses could be performed.

Only Ghendon et al. [25] reported on pneumonia, and found a significant reduction in contacts of influenza vaccinated patients (OR: 0.38; 95% CI: 0.30–0.50). Hospital admission was only reported in one study [14]; showing higher hospital admission rates in contacts of vaccinated persons (OR: 1.92; 95% CI: 1.17–3.14). There were no studies reporting on mortality endpoints.

### Risk of bias and grading of evidence

The most common potential risks of bias in the included RCTs were lack of appropriate generation of the randomisation sequence, lack of allocation concealment and lack of blinding of patients and healthcare providers (Table 2). The RCTs scored a mean of 4.3 (range: 2–7) when assessed against seven domains. 

**Table 2:** Risk of bias in nine included randomised controlled trials reporting on influenza infections in contacts of influenza vaccinated vs unvaccinated individuals in non-healthcare settings

First author [source]	Risk of bias						
	Sequence generation	Allocation concealment	Blinding of patients	Blinding of healthcare provider	Blinding of outcome adjudicators	Incomplete data addressed	Selective reporting
Gruber [29]	NK	NK	Low	Low	Low	Low	Low
Clover [33]	NK	NK	Low	NK	Low	Low	Low
Rudenko [23]	NK	NK	Low	NK	Low	Low	Low
Hurwitz [13]	NK	NK	Low	NK	NK	NK	Low
Esposito [34]	Low	NK	Low	Low	Low	Low	Low
Principi [24]	NK	NK	High	High	NK	Low	Low
Hui [31]	NK	NK	High	High	Low	Low	Low
Cowling [30]	Low	NK	Low	Low	Low	Low	Low
Loeb [12]	Low	Low	Low	Low	Low	Low	Low
Percentage low risk of bias^a^	33	11	22	33	78	89	100

NK: not known, as either unclear or not reported.

^a^ The percentage low risk of bias for each domain was calculated by dividing the number of randomised controlled trials (RCTs) at low risk of bias by the total number of RCTs (n = 9).

The observational studies were awarded a mean of 6.25 points of a maximum of nine on the Newcastle-Ottawa scale, i.e. they were in a middle range of risk of bias (7 for Piedra et al. [26] and Ghendon et al. [25], 6 for Kjos [27] and 5 for King et al. [14]).

Applying GRADE criteria, we decreased the level of evidence for the primary outcome because of serious limitations in the quality of the studies (i.e. risk of bias in RCTs and observational design in non-RCTs) and inconsistency with significant statistical heterogeneity. Therefore, the overall level of evidence supporting a herd effect of influenza vaccines in preventing influenza virus infection in contacts in non-healthcare settings was considered to be low.

## Discussion

We found an overall low level of evidence supporting an indirect or herd effect of influenza vaccination in preventing influenza virus infection in vaccinated persons’ contacts. In all but one study we identified, children were vaccinated. While observational studies showed a significant effect, the summary estimates from RCTs did not show a statistically significant effect. Few data were available on herd effect of influenza vaccination preventing hospital admission, pneumonia and death.

Point estimates of four of the six RCTs that reported on the prevention of influenza virus infection in contacts of vaccinated persons pointed towards a potential benefit of vaccination, but no significant effect was found overall. In an RCT by Loeb et al. involving Hutterite communities [12], vaccination of children in an enclosed community significantly reduced influenza infections in contacts. The uptake of influenza vaccination in that RCT, which had a low risk of bias in all domains assessed, was ca 83%. The RCT confirmed the findings from an observational study by Monto et al. that found a similar effect at the population level by vaccinating schoolchildren in one community in Michigan, United States [28]. However, no strong evidence was found in a household setting [29,30]. A possible explanation is that vaccinating only one child per household, as done in the study by Cowling et al., may have been insufficient to have a measurable effect [30]. In the study by Gruber et al., in contrast, all children three years of age and older received the vaccine, but again there was no effect on household contacts. However, the study was limited by the low attack rate and was therefore likely underpowered [29]. Furthermore, the authors argued that the non-vaccinated contacts were likely to be immune to the predominant influenza B strain that circulated in previous years. It is therefore unclear what key factors are needed to achieve a herd effect in the household, particularly given the importance of the broader community as a potential source of infection of the non-vaccinated. Notably, the only study that investigated herd effect of influenza vaccination of adults did find a statistically significant effect [31]. However, this study had significant methodological limitations, including lack of blinding. It should be acknowledged that two studies that both reported a significant herd effect of influenza vaccination could not be included in the meta-analysis because of the lack of detail reported in the published article, and no additional information could be obtained from the authors [23,24]. 

In contrast to our findings from RCTs, we found evidence of herd effect following influenza vaccination in observational studies, which was corroborated by a recent observational study by Pannaraj et al., who found that unvaccinated children may be protected in schools with vaccination rates approaching 50% [32].

Our extensive screening of over 40,000 studies found very few studies that were designed to measure herd effects of influenza vaccination. One reason for this may be the cost of community influenza surveillance as well as the cost of clinical trials. While modelling studies demonstrate that herd immunity can be achieved by vaccinating young children [10], we are surprised by how few studies with laboratory-confirmed influenza as an outcome support the modelling literature. Moreover, there are very limited data available to estimate herd effect of influenza vaccination programmes. As indirect benefits would increase the cost-effectiveness of these programmes, such data would be highly valuable for vaccine advisory bodies and decision makers evaluating whether to initiate or expand influenza vaccine programmes.

Our review highlights the need for more rigorous studies using laboratory-confirmed influenza virus infections as an outcome. Data on a herd effect on outcomes other than influenza virus infection were sparse, due either to outcomes not being measured or to inadequate power to detect a difference. Although the effect of influenza vaccination on mortality has been demonstrated through modelling [10], high-quality studies would better support the ability of influenza vaccination to prevent hospital admissions, pneumonia or death in contacts through herd effect.

Strengths of this systematic review include a systematic, protocol-driven and comprehensive review with extensive literature search strategy including RCTs and observational studies. In addition, rigorous assessment of eligibility ensured high reliability of the results. All subgroup analyses were defined a priori. A rigorous use of the GRADE approach ensured a transparent and comprehensive approach to evaluate overall quality of the studies. An important limitation, however, was the presence of statistically significant heterogeneity that could not be explained by a priori defined subgroup analyses. We assume that differences in study designs and clinical heterogeneity in terms of study population, outcome assessment and health service resources may have resulted in differences in outcomes that could not be explained by the intervention per se. Furthermore, differences in vaccine effectiveness in case of mismatch and existing immunity if the circulating strain had been dominant for several seasons may have introduced heterogeneity across the included studies. Another major limitation was the potential risk of bias in the majority of studies, which further decreased the level of evidence. Finally, all but one study vaccinated children, thus, no generalisation to vaccination programmes in adults can be made, and the evidence is too limited to conclude in what setting(s) a significant herd effect may or may not be achieved.

In summary, herd effects are assumed with influenza vaccine programmes, but there are few studies that quantify the herd effect of vaccination. We found low-level evidence supporting a herd effect of vaccination on influenza virus infection in contacts of vaccinated persons. Further rigorous studies are needed in order to better understand under which circumstances vaccination may prevent influenza and its complications in contacts.
